# Acknowledgment to Reviewers of *Journal of Fungi* in 2020

**DOI:** 10.3390/jof7020072

**Published:** 2021-01-20

**Authors:** Journal of Fungi Editorial Office

**Affiliations:** MDPI AG, St. Alban-Anlage 66, 4052 Basel, Switzerland

Peer review is the driving force of journal development, and reviewers are gatekeepers who ensure that *Journal of Fungi* maintains its standards for the high quality of its published papers. Thanks to the cooperation of our reviewers, in 2020, the median time to first decision was 16 days and the median time to publication was 33 days. The editors would like to express their sincere gratitude to the following reviewers for their precious time and dedication, regardless of whether the papers were finally published:
A.Yu. Vasil’kov, AlexanderLopez-Ribot, JoseAbd-Elsalam, Kamel A.Lorenz, AlexanderAbd-Elsalam, Kamel AhmedLucero, Leandro ExequielAbdolrasouli, AlirezaM. Caramella, CarlaAbel-Santos, ErnestoMachado, HelenaAbu-Saleh, Omar M.Macias Sanchez, Antonio JoséAdamkova, VaclavaMacioszek, ViolettaAgustinho, DanielMacirella, RacheleAimanianda, Vishu KumarMaddi, AbhiramAkira, YoshimiMahmoud, MedhatAlam, MahboobMajoros, LaszloAlanio, AlexandreMang, Stefania MirelaAlberti, FabrizioMankowski, Mark E.Albuquerque, PatríciaMantadakis, ElpisAlonso-Monge, RebecaMantzoukas, SpiridonAlsibai, Kinan DrakMarcos, CarolineAlves, ArturMareković, IvanaAmillis, SotirisMares, MihaiAmmirati, Joseph F.Marocco, AdrianoAnand, RuchikaMarques, Maria RitaAnanda-Rajah, MichelleMartău, Adrian GheorgheAnastasopoulou, AmaliaMartin, KirchmairAndersen, BirgitteMartinez-Montemayor, MichelleAnderson, MatthewMartin-Gomez, M.TeresaAndes, DavidMartini, CeciliaAndré, BrunoMartins, IsabelAndrianaki, AngelikiMartins, NatáliaAoki, TakashiMartin-Vicente, AdelaAppell, MichaelMaruthamuthu, MuraliArabatzis, MichaelMarzban, GorjiAraniciu, CătălinMasaphy, SegulaArastehfar, AmirMastore, MaristellaArendrup, MaikenMatharu, NavneetArsenijević, Valentina ArsićMatny, OadiAttanayake, Renuka N.Matoulková, DagmarAzar, Marwan MMatsuda, YudaiAzevedo, JorgeMatthijs, SeverineBąchor, RemigiuszMay, TomBader, OliverMayser, Peter AndreasBae, YoungjuMazur, AndrzejBahr, Nathan CMccarthy, PeterBailón-Ruiz, Sonia JanetMcClelland, ErinBairwa, GauravMcKenzie, EricBaj, JacekMcKinstry, Karl KaiBajaj, RuchikaMcMurray, MichaelBaldin, ClaraMead, Matthew E.Balestrini, Raffaella MariaMeijer, Eelco F.J.Ballinas, LourdesMeis, Jacques F.Bar, MayaMelchers, WillemBarantsevich, E.P.Mellado, EmiliaBarber, AmeliaMello, AntoniettaBarker, BridgetMendes-Giannini, Maria José SoaresBaswan, SudhirMendling, WernerBates, StevenMendoza, ArtemioBecchimanzi, AndreaMenon, AnantBecker, PierreMensah-Attipoe, JacobBedre, ReneshMercier, ToineBelbahri, LassaadMešić, ArminBelfiori, BeatriceMesini, AlessioBen-Ami, RonenMestrovic, TomislavBennett, Richard J.Mezule, LindaBensch, KonstanzeMierzejewska, JolantaBerger, Ralf G.Mihai, MaraBernal-Martínez, LeticiaMira, Nuno PereiraBernat, PrzemysławMiranda, IsabelBertea, CinziaMiranda, LuisaBi, YangMitsuyama, SusumuBiernasiuk, AnnaMiura, ChihiroBisko, Nina A.Miura, NatsukoBłaszczyk, LidiaMohapatra, Bidyut R.Blauth De Lima, FernandaMoldovan, Radu-CristianBliss, Joseph MMołoń, MateuszBobbo, TaniaMonk, BrianBoerner, G. ValentinMonteoliva, LuciaBombarda, IsabelleMoore, Sean D.Bonfante, PaolaMorales, DiegoBongomin, FelixMora-Montes, HectorBonvicini, FrancescaMoran, GaryBorman, Andrew M.Mori, KoichiroBoruta, TomaszMorio, FlorentBörve, JorunnMorris, Arthur J.Botterel, FrançoiseMuggeo, PaolaBravo, ArmandoMuntyan, Maria SBrígido, ClarisseMuszewska, AnnaBrillowska-Dąbrowska, AnnaMuszyńska, BożenaBrown, PaulMutiga, Samuel K.Brüggemann, Roger J.Nacher, MathieuBrun, SophieNai, Yu-ShinBrun, SylvainNakamura, TaroBrunke, SaschaNakayasu, ErnestoBruno, VincentNakazawa, TakehitoBryan, ArielleNanjappa, Som G.Bryła, MarcinNarcisa, MandrasBuchheidt, DieterNarra, Hema PrasadBui, Duc-CuongNatvig, Donald O.Buil, Jochem B.Navarro-Mendoza, MariaBuitrago, María JoséNawaz, AliBujdáková, HelenaNawrot, UrszulaBurmester, AnkeNelsen, Matthew P.Burrack, LauraNermut, JiříBusca, AlessandroNerva, LucaBuscaino, AlessiaNesterov-Mueller, AlexanderBuzina, WalterNeubauer, DamianC. J. M. D. S. Menezes, JoséNicola, AndréCacciola, Santa O.Nicolas, Francisco E.Cacciola, Santa OlgaNicoletti, RosarioCaceres, Diego HNiemiec, Maria JoannaCacho Teixeira, MiguelNikolaivits, EfstratiosCai, LeiNile, ChristopherCal, Antonieta DeNilsson, HenrikCal, MagdalenaNobile, Clarissa J.Calderone, RichardNordøy, IngvildCalich, Vera Lucia GarciaNosanchuk, JoshuaCamenzind, TessaNosanchuk, Joshua D.Campos, Maria DoroteiaNovak-Frazer, LilyAnnCannon, RichardNovotna, AlzbetaCao, HuanshengNowicki, RomanCapozzi, VittorioNowrousian, MinouCarpentier, FantinNutzmann, Hans-WilhelmCarqueijeiro, InêsO.Keyhani, NematCarrillo, WilmanObar, Joshua J.Carvalho, AgostinhoObayashi, TaminoriCarvalho, Lina A.S.Ogawa, MasahiroCasadevall, ArturoOgihara, JunCastella, GemmaOgórek, RafałCastro-Lopez, NataliaOh, Seung-YoonCatania, Maria RosariaOkorski, AdamCattaneo, ChiaraOlenin, Andrei YuCenci, ElioOliveira Souza, Ana CamilaCerqueira, LauraOliveira, ManuelaCesaro, SimoneOlszewski, Michal AChakankar, MitalOniga, Smaranda DafinaChamper, JacksonOrasch, ThomasChandrasekar, PranatharthiOrihara, TakamichiChandrasekaran, MurugesanOsei-Amponsah, RichardChang, Cheng-Wei TomOsherov, NirChang, Yun-TsanOsivand, AsmaChapeland-Leclerc, FlorenceOtašević, SuzanaChaturvedi, VishnuOtero-Espinar, Francisco JavierChauhan, NeerajOtulak-Kozieł, KatarzynaChaves, Susana RodriguesOzaki, TaroChen, Chih-ChiehÖzenci, VolkanChen, FushengPadoin, ChristopheCheng, Yi-ShengPagano, LivioChilders, Delma SusanPagano, LucaChoi, Ki ChoonPage, StephenChow, JackyPagniez, FabriceCitores, LuciaPais, PedroCohen, RonPan, YoulianColeine, ClaudiaPark, Young-JinColeman, JeffreyPârvu, MarcelColombo, ArnaldoPathirana, Ruvini U.Colosi, Ioana AlinaPatyra, EwelinaConti, StefaniaPavlidis, Ioannis V.Cook, PeterPawlikowska-Pawlęga, BożenaCordovana, MiriamPearce, CedricCormack, BrendanPecka-Kiełb, EwaCorreia, InêsPedrini, NicolásCorry, David B.Pennerman, KaylaCorzo-León, Dora E.Pérez García, Luis AntonioCosta, StefaniaPerez-Garcia, AlejandroCosta-de-Oliveira, SofiaPerez-Martin, JoseCostantini, ClaudioPeric, MirjanaCouppié, PierrePerlin, Michael H.Courty, Pierre-EmmanuelPeterson, MichaelCovo, ShayPetraitis, VidmantasCraig, MorriePfaller, Michael A.Creamer, RebeccaPfliegler, Walter P.Crouch, KathrynPieckova, ElenaCruciani, MarioPietras, MarcinCutrignelli, Monica IsabellaPiłsyk, SebastianCytryńska, MałgorzataPimentel Barros, PatríciaDa Silva, Isabel João SoaresPinto, EugéniaDai, JiangkunPla, Jésus C.Daniela, JakšićPlacido, JerssonDannaoui, EricPoggi, AlessandroDantas, Alessandra Da SilvaPolčic, PeterDatta, KausikPoldmaa, KadriDavidsen, Jesper RomhildPoli, AnnaDavis, JamesPolizzi, GiancarloDe Bellis, LuigiPollmann, KatrinDe Biasi, Margherita G.Ponce-Vargas, MiguelDe Castro Keller, AlexandrePop, Oana LeliaDe Cock, HansPopolo, Laura MariaDe Macedo, Priscila MarquesPortilla, MaribelDe. Hoog, Sybren G.Posteraro, BrunellaDebuchy, RobertPotekhin, AlexeyDeepe, GeorgePoussereau, NathalieDegani, OfirPoyntner, CarolineDegola, FrancescaPrattes, JuergenDel Campillo, Maria CarmenPrice, PatriciaDellière, SarahPrieto, DanielDesai, JigarPurdy, JohnDesnos-Ollivier, MariePuri, SumantDhaundiyal, AlokPyka, AlinaDi Gennaro, FrancescoQueiroz-Telles, FlavioDi Michele, AlessandroRachel Basques, CaligiorneDi Piazza, SimoneRadim, DobiášDiamond, GillRafiq H Siddiqui, MohammedDíaz, JoséRaisanen, RiikkaDichtl, KarlRajarapu, Swapna PriyaDillman, Adler R.Rajput, Vishnu D.Dimopoulos, GeorgiosRamamoorthy, AyyalusamyDimopoulou, MariaRamírez Soto, Max CarlosDirk, FriedrichRamirez-Garcia, AndoniDogra, PranayRamón R., BarralesDolan, StephenRamonell, KatrinaDonadu, Matthew GavinoRapoport, AlexanderDonati, IreneRappleye, ChadDreaden, Tyler J.Rauceo, Jason M.Drummond, Rebecca A.Rébé, CédricDuan, PuReed, Kevin F.M.Dubovskiey, IvanRehrig, ErinDufossé, LaurentReichner, JonathanDufour, Bernard P.Reis, CristianoDufresne, PhilippeRementeria, AitorDylag, MariuszReumamm, SigrunEbel, FrankReuter, KlausEbrahim, WeaamRezania, ShahabaldinEdgerton, MiraRibes, JulieEgito, Eryvaldo Socrates Tabosa DoRickerts, VolkerEk-Ramos, Maria JulissaRinaldi, Andrea C.Elad, DanielRivero-Menendez, OlgaEl-Esawi, Mohamed A.Ro, Hyeon-SuEliopoulos, Panagiotis A.Robbertse, BarbaraEllervik, UlfRobert-Gangneux, FlorenceElsie, Parés-MatosRobinson, SeriEmpting, MartinRodrigo, Sara MoralesEndoh, RikiyaRodrigues, Célia F.Erdman, ScottRodrigues, Fernando José SantosErnesto Vidal, JoséRodriguez De La Vega, RicardoEscandón, PatriciaRodriguez Vazquez de Aldana, BeatrizEscribano, PilarRogers, ThomasEsher, ShannonRogozhin, EugeneEspeso, Eduardo A.Roilides, EmmanuelEspinel-Ingroff, AnaRollins-Smith, LouiseEspinel-Ingroff, Ana V.Romo, JesusF. Abdallah, MohamedRonda, LucaFaergemann, JanRoriz, MarianaFarr, AlexRosenau, FrankFasciana, TeresaRoslin, TomasFathi Abdallah, MohamedRosowski, EmilyFathollahzadeh, HomayounRossoni, Rodnei DennisFaulds, CraigRostislav, ZemekFerrante, ClaudioRuano, FranciscaFerrara, MicheleRühl, MartinFerrigo, DavideRurack, KnutFidel, Paul L.Russo, AlessandroFlanagan, DennisRuytinx, JoskeFlores-Félix, J.D.Sabino, RaquelFontaine, ThierrySacedón, RosaFontana, FabrizioSakai, HiromiForche, AnjaSakurai, AkihikoForsberg, KaitlinSalzano, Anna MariaFree, StephenSamonis, GeorgeFriaza, VicenteSampaio, PaulaFunari, ValerioSanae, Ishijima A.Gacser, AttilaSánchez Crespo, MarianoGaglione, RosaSanchez, YolandaGago, SaraSanglard, DominiqueGajger, Ivana TlakSantos, CarlaGallotti, FrancescaSantos, JoséGálvez Buerba, Eva M.Santos, LilianaGangneux, Jean PierreSarasan, ViswambharanGarbrecht Thygesen, LisbethSarrocco, SabrinaGarcia-Rubio, RocioSass, GabrieleGarrido-Jurado, InmaculadaSatake, MasayukiGeng, HuilingSaunte, DitteGenovese, CarloSavoie, JeanGhannoum, MahmoudSawbridge, Timothy I.Ghosh, ArnabScandellari, FrancescaGiannesi, Giovana C.Schalk, EnricoGigliotti, FrancisSchauwvlieghe, Alexander F.A.D.Giordani, BarbaraScherm, HaraldGiordano, MariaSchipper, KerstinGioti, AnastasiaSchmidt, OlafGirometta, Carolina ElenaSchmidt, StanislawGits-Muselli, MaudSchriawski, JanGladstone, SilvaSchrödl, WielandGlazer, ItamarSchüller, ChristophGlazunova, Olga A.Schwartz, IlanGnat, SebastianSchwartz, StefanGodoy, JayfredSchwarz, PatrickGoldmann, KeziaSchweigkofler, WolfgangGomoiu, IoanaŠeda, MartinGonçalves, A PedroSee, Pao TheenGonda, SándorSegal, EstherGONGAL, GyanendraSeok, HyeriGontikaki, EvinaSeybold, UlrichGonzález-Lara, María FernandaSHAFIGH, PAYAMGoodman, Alan G.Shakya, Viplendra P.S.Gow, NeilShanmugasundram, AchchuthanGrabner, Daniel S.Sharma, CheshtaGreb-Markiewicz, BeataSharp, JoGresnigt, MarkShaw, SophieGrieco, FrancescoShekhova, ElenaGroll, AndreasShetty, Kateel G.Gromadzka-Ostrowska, JoannaShigeyuki, KawaiGuarnaccia, VladimiroShilman, Mikhail MartchenkoGube, MatthiasShin, Kwang-SooGuenova, EmmanuellaShor, ErikaGuirao-Abad, José P.Silar, PhilippeGutiérrez Serpa, AdriánSimm, ClaudiaGutierrez, SantiagoSingh, ReemaGuzik, MaciejSingh, ShaktiHaas, HubertusSipos, GyörgyHagiwara, DaisukeŠirić, IvanHahn, MatthiasSkaltsa, LianaHamada, YukihiroSkiada, AnnaHameed, KhalidSkrypnik, KatarzynaHammerschmidt, RaySnelders, EvelineHančević, KatarinaSoares, Celia Maria De AlmeidaHaneke, EckartSokovic, MarinaHao, GuixiaSolano, FranciscoHartung, JensSolcan, CarmenHashimoto, NaotoSomma, StefaniaHassan, Tidi MaharaniSong, HaoHay, RoderickSong, YuandaHayashi, NagaoSorrell, Tania C.Heber, DavidSossidou, EvangeliaHeczko, PiotrSpakowicz, DanHeinisch, JürgenSparks, MichaelHeinz, WernerSpecht, Charles AHeitman, JosephSpeth, CorneliaHelden, MaartenSrivastava, Dhruv AdityaHennequin, ChristopheStączek, SylwiaHennicke, FlorianStana, AncaHerbert, De’Broski R.Stanford, StephanieHernández, María Del MarStaniszewska, MonikaHernández-León, AlejandroStaszczak, MagdalenaHernández-Montiel, Luis GuillermoStaver, CharlesHettiarachchige, Inoka K.Steel, Christopher C.Heung, LenaSteinmann, JoergHigo, MasaoStines-Chaumeil, ClaireHoebe, PeterSturtevant, JoyHoenigl, MartinSuarez-Trujillo, AridanyHof, HerbertSugumaran, ManickamHohmann, StefanSultana, HalimaHolb, ImreSun, XiangHolbech Jensen, Benjamin AnderschouSwidergall, MarcHolsinger, DamainSzweda, PiotrHomma, TetsuyaTachikawa, HiroyukiHong, Chang PyoTagliavia, MarcelloHooykaas, PaulTailhades, JulienHospenthal, Duane R.Taíssa Vieira Machado, VilaHuang, LindaTakenaka, MotoiHvattum Divon, HegeTalento, AlidaHyrsl, PavelTalhinhas, PedroIbarra, DavidTarasco, EustachioIkeda, KenichiTatsumi, YoshiyukiImhoff, JohannesTavanti, AriannaInfascelli, FedericoTen Oever, JaapInokuchi, TsutomuTerhonen, EevaIoannou, PetrosTharreau, DidierIonut, Ioana A.Thekkiniath, JoseIrie, ToshikazuThevissen, KarinIwata, AtsushiThipe, Velaphi C.Iwona, WojdaThomas Shier, WayneJenks, Jeffrey D.Tian, MiaoyingJewell, Jeremy B.Timón-Gómez, AlbaJia, LeijieTorres, NazarethJia, MelissaTóth, ZoltánJiang, YuemingTragiannidis, AthanasiosJiménez-Ortigosa, CristinaTraven, AnaJohansen, AndersTreu, RolandJohnson, ElizabethTripathi, Siddharth KaushalJosue, DelgadoTsai, Catherine Jia-YunJunqueira, JulianaTsai, Ming-HorngJunqueira, Juliana CamposTuovinen, OlliJuwadi, Praveen RaoTurkoglu, AzizKalkum, MarkusTwarużek, MagdalenaKamińska, JoannaUehling, Jessie KKaraman, MajaUnković, NikolaKarcz, DariuszUrbaniak, MonikaKarisola, PiiaUrlich, SebastianKarki, BishnuUsher, JaneKarkowska-Kuleta, JustynaUzunhan, YurdagulKarpe, Avinash V.Valentin, ThomasKauffman, CarolValiante, VitoKawasaki, HideyaVan Braeckel, EvaKayumov, AiratVan Der Nest, Magriet AKazimierczak, WaldemarVan Dijk, KarinKean, RyanVan Wuytswinkel, OlivierKeniya, Mikhail V.Varcamonti, MarioKhan, NaeemVargas, SergioKim, JaehanVasconcelos, MartaKim, JoonhoonVatanshenassan, MansourehKim, SoonokVeloso, JavierKioshima, Erika SekiVenkannagari, HariKanthKitagaki, HiroshiVergidis, PaschalisKiyomi Yamakawa, CelinaVernelen, KrisKlaile, EstherVernero, MartaKlempová, TatianaVestergaard, Mun’delanji C.Klimek-Ochab, MagdalenaVicentefranqueira, RocíoKlimko, NikolaiVideira, ArnaldoKnezevic, JelenaVidican, RoxanaKobae, YoshihiroViegas, CarlaKoehler, PhilippVieira Loures, FlávioKoide, Roger T.Vita, FedericoKolls, Jay K.Vlahovic, TraceyKolomytseva, MarinaVoglmayr, HermannKong, Won-SikVon Zeska Kress, Márcia ReginaKonno, NaotakeVylkova, SlavenaKonopka, JamesVyzantiadis, Timoleon-AchilleasKonopka, James B.Wada, GloriaKontogiannatos, DimitriosWagner, LysettKorac, AleksandraWaldman, BruceKordalewska, MilenaWalker, Louise A.Kornitzer, DanielWall, GinaKosakyan, AnushWalther, GritKosmidis, ChrisWANG, CHENGSHUKotta-Loizou, IolyWang, Chih-LiKovacs, RenatoWang, Hong-KaiKovács-Öller, TamásWang, ShihuaKozłowska, JoannaWang, TuoKrnjaja, VesnaWang, YanKruszewska, JoannaWang, YufengKrysan, DamianWarris, AdiliaKryukov, Vadim YWatanabe, AkiraKucharíková, SoňaWatkins, TonyaKuchler, KarlWebb, GinnyKuhnert, EricWellinger, Ralf-ErikKumar, AnandWendland, JürgenKumar, ManuWengenack, NancyKumar, RohitashwWhite, LewisKumaresan, PappanaickenWiederhold, NathanKunicka-Styczyńska, AlinaWiewióra, BarbaraKupfer, AlexanderWillaert, RonnieKurzai, OliverWillems, Hubertine M. E.Kuźniar, AgnieszkaWilliamson, PeterKyriakidis, IoannisWilson, BreeL. McCulley, RebeccaWolff, Anette Susanne BøeLachance, Marc-AndréWolina, UweLackner, MichaelaWolny-Koładka, KatarzynaLagrou, KatrienWong, Sarah Sze WahLake, Douglas F.Woods, Jon P.Lalak-Kańczugowska, JustynaWöstemeyer, JohannesLamping, ErwinWright, Terry W.Lanternier, FannyWu, Shu-JuLapaquette, PierreWurster, SebastianLarena, InmaculadaWyszkowska, JadwigaLarkin, Paige M. K.Xing, FuguoLatgé, Jean-PaulXu, JianLavergne, Rose-AnneXu, JianpingLeal, FernandoXue, ChaoyangLeber, ReginaYadav, Narendra SinghLee, Cheng-IYamamoto, NaomichiLee, Marcia R.Yamanaka, TakashiLee, Mark J.Yang, ShangxinLee, Soo ChanYano, JunkoLeibundGut-Landmann, SaloméYu, ShenLeitão, AnaYun, Cheol-WonLeón, MaelaYurchenko, EkaterinaLepak, AlexanderYurkov, Andrey P.Leslie, Jhansi L.Zaias, NardoLevy, StevenZajc, JanjaLewis, Russell EdwardZanetti, StefaniaLi, GangZarei Mahmoudabadi, AliLiao, Chien-SenZavarzina, AnnaLin, Ching-HsuanZervakis, Georgios I.Lin, XiaorongZhang, HongyinLinaldeddu, Benedetto T.Zhang, Jing-ZeLingscheid, TilmanZhong, JieLipke, PeterZhuo, ChunliuLitvintseva, Anastasia P.Zhuravleva, Olesya I.Liu, JinxinZielinski, ChristinaLo Giudice, AngelinaZifcakova, LuciaLockhart, Shawn R.Zimmermann, Sabine DagmarLoh, JacelynZNAIDI, SadriLombardi, LisaZoll, JanLopes, GracilianaZrenner, Rita MariaLopez-Llorca, Luis V.Zucconi, Laura

